# On the Physical Stability of Leucine-Containing Spray-Dried Powders for Respiratory Drug Delivery

**DOI:** 10.3390/pharmaceutics15020435

**Published:** 2023-01-28

**Authors:** Mani Ordoubadi, Kimberly B. Shepard, Hui Wang, Zheng Wang, Amanda M. Pluntze, Joseph P. Churchman, Reinhard Vehring

**Affiliations:** 1 Department of Mechanical Engineering, University of Alberta, Edmonton, AB T6G 2E1, Canada; 2 Research & Development, Lonza Group AG, Bend, OR 97703, USA; 3 Product Development, Lonza Group AG, Bend, OR 97703, USA

**Keywords:** spray drying, L-leucine, dry powder inhalers, fibers, powder stability

## Abstract

Carrier-free spray-dried dispersions for pulmonary delivery, for which the demand is growing, frequently require the incorporation of dispersibility-enhancing excipients into the formulations to improve the efficacy of the dosage form. One of the most promising of such excipients, L-leucine, is expected to be approved for inhalation soon and has been studied exhaustively. However, during stability, small fibers protruding from the particles of leucine-containing powders have occasionally been observed. To clarify the origin of these fibers and assess their potential influence on the performance of the powders, three different classes of spray-dried leucine-containing formulation systems were studied over an 8-month accelerated stability program. These systems consisted of a large molecule biologic (bevacizumab) in conjunction with a glass former (trehalose), an amorphous small-molecular mass active (moxidectin), and a crystallizing active (mannitol). It was determined that the appearance of the fibers was due to the presence of small quantities of leucine in higher energy states, either because these were amorphous or present as a less stable crystalline polymorph. It was further shown that the growth of these leucine fibers caused no significant physicochemical instability in the powders. Nor, more importantly, did it decrease their aerosol performance in a dry powder inhaler or reduce the concentration of their active pharmaceutical ingredients.

## 1. Introduction

Spray drying is a particle engineering technique that is used to manufacture respirable powders for inhaled delivery to the deep lung. Key attributes for successful delivery to the bronchi, bronchioles, and alveoli within the lung include a suitable aerodynamic particle size [1,2] and optimal dispersibility of the powders [3,4]. When delivered by a passive dry powder inhaler, about 1 to 50 mg of powder, typically contained in a capsule or blister, is dispersed by the patient’s own breath. Cohesive or poorly dispersible powders may not deagglomerate to their primary particle size, causing some of the material to remain in the DPI device, or to deposit at the back of the throat. Dosing variability resulting from inadequate aerosolization properties must be avoided for a successful inhalation product. These challenges to achieving the desired product quality attributes require a careful mechanistic design of the process and formulation to overcome them.

The formulation design of spray-dried pulmonary particles depends on the type of active ingredient, which can be categorized into three classes: (1) small-molecule drugs that tend to crystallize during spray drying, (2) small-molecule drugs that tend to dry into a glass, and finally, (3) large macromolecular biologics that are generally amorphous after spray drying [5]. Depending on the class of the drug, different excipients have frequently been included in formulations to improve the physicochemical properties and efficacy of the carrier-free product [4]. For example, glass stabilizers with high glass transition temperatures, such as trehalose, have been added to formulations, both to increase the storage stability of the powders and to reduce the risk of processing damage to biologics, such as proteins [6,7]. As opposed to the more conventional micronized carriers, these excipients are spray-dried together with the actives and hence, undergo simultaneous particle formation resulting in complex particles.

Because amorphous particles tend to be relatively cohesive and hence, disperse poorly during delivery via dry powder inhalers [3,8], dispersibility enhancers have often been used to reduce the inter-particle cohesion in powders in order to improve the delivered dose and the final efficacy of the product [9]. L-leucine is one such dispersibility enhancer that is frequently used in the spray drying of particles for respiratory delivery to the lungs [7,10,11,12]. Its positive impact in improving the aerosol properties of spray-dried inhalation powders has been extensively reported [13,14,15]. Leucine is surface-active [16], has a relatively low aqueous solubility of ~22 mg/mL [17], and tends to crystallize during spray drying [18]. All of these properties make leucine an ideal dispersibility enhancer, due to its ability to form a crystalline shell on the surface of the spray-dried particles that reduces surface energy while increasing surface rugosity [13]. It has been explained elsewhere that leucine instantaneously nucleates and grows crystals on the surface of evaporating droplets once its local concentration reaches a critical supersaturation ratio, which can be predicted [10]. This information can be used in the formulation design of leucine-containing systems to achieve the goal of a fully crystalline shell on the surface of the particles for maximum effectiveness [4].

The stability of spray-dried particles containing leucine has been investigated previously, with the understanding that crystalline excipients are, in general, physically stable, especially if stored in dry conditions [11,13,19,20,21]. However, even in properly designed leucine-containing multi-component particles, a small amount of leucine may remain amorphous after drying, and the resulting leucine shell has occasionally been found to consist of a large number of nanocrystals [10]. A fraction of the leucine may therefore not be in its lowest energy state, corresponding to macroscopic crystals consisting of the most stable polymorph. Given sufficient mobility, this fraction of leucine may have the potential to convert into structures with lower energy, e.g., larger or newly formed crystalline structures, such as fibers.

In this study, we aimed to investigate the frequency of appearance and the formation mechanisms of such fibers in spray-dried particles, and to study whether they have any meaningful effect on aerosol performance and the overall physicochemical stability of the powders. To this end, three model systems of interest with leucine as the shell former were selected: moxidectin as a small-molecule drug that does not crystallize during spray drying, mannitol as a small-molecule active that does crystallize upon drying, and bevacizumab as a biologic that is always amorphous after spray drying. To study the effect of leucine’s solid phase on fiber formation and its effect on product performance, three formulations with varying levels of leucine crystallinity were designed for each system, with the help of advanced particle formation models.

## 2. Materials and Methods

### 2.1. Materials

Materials consisted of L-leucine with a purity exceeding 98.5% (Cat. No. 208306, J.T. Baker, Phillipsburg, NJ, USA), trehalose dihydrate (Cat. No. T-104-4x, Pfanstiehl, Waukegan, IL, USA), D-Mannitol (Cat. No. 1.00419, Millipore Sigma, St. Louis, MO, USA), and moxidectin (Cat. No. 319514, MedKoo Biosciences, Morrisville, NC, USA). Bevacizumab was obtained as a sterile aqueous solution in 50 mM phosphate buffer (pH 6.2) with 30 mg/mL bevacizumab, 60 mg/mL trehalose, and 0.04% *w*/*w* polysorbate 20.

### 2.2. Methods

#### 2.2.1. Theoretical Formulation Design

Advanced particle formation models [22,23] were used to design three different formulations for each of the three model systems with the aim of achieving different leucine crystallinity levels, ideally spanning from 0% to 100%. Through this approach, the effect of partial leucine crystallinity on storage stability could be examined in detail.

During spray drying, depending on their plasticized behavior, glass formers undergo either liquid–liquid or glass–liquid phase separation upon reaching their amorphous solubilities [24,25,26]. In contrast, crystallizing compounds are expected to start nucleation and crystal growth after reaching certain levels of supersaturation [27]. Further increase in supersaturation decreases the delay in nucleation to the point at which it can be assumed to be instantaneous, termed critical supersaturation [28]. Given the very fast rates of evaporation encountered during spray drying, this critical supersaturation has previously been used to explain the particle formation of leucine in aqueous and cosolvent systems [10,29]. Based on this information, a critical concentration was determined for each formulation component, depending on its expected solid phase. These critical concentrations were then compared to their instantaneous surface concentrations during droplet evaporation to determine the sequence of solidification for all components. The surface concentrations of the compounds were calculated using a numerical model for both single-solvent and multi-solvent systems, as explained in detail elsewhere by Ordoubadi et al. [22]. Taking into account the distinct mechanisms of solidification in each of the three systems, the formulation design was performed as discussed below. In the numerical models, an approximate drying temperature of 70 °C, a value that falls between the chosen inlet and outlet conditions, was selected to take into account the temporal and spatial variation of temperature in the drying chamber of the lab-scale spray dryer.

##### Bevacizumab–Trehalose–Leucine System in Water

This aqueous system contained a macromolecular glass former (bevacizumab), a small-molecule glass former (trehalose), and a crystallizing shell former (leucine). For such a system containing a monoclonal antibody, trehalose as a glass-forming excipient is intended to provide stabilization [9]; hence, the active-to-glass former ratio is an important parameter to consider, both in terms of increasing the glass transition temperature and of the stabilizing protein. In previous work, three formulations with mass fractions of 0.1/0.7/0.2, 0.2/0.6/0.2, and 0.4/0.4/0.2 bevacizumab/trehalose/leucine were all found to be physically stable at room temperature [7]. Here, an intermediate bevacizumab-to-trehalose mass ratio of 1:4 was chosen. A reason for selecting this constraint was to have small enough fractions of the protein in the system to be able to design the formulation with high leucine crystallinity, as high protein mass fractions can interfere with the crystallization of other components [10].

With the help of numerical predictions [22] based on a total feed concentration of 40 mg/mL, and under the constraint set for the bevacizumab-to-trehalose ratio, different mass fractions of leucine were calculated to cover the intended leucine crystallinity span. For leucine to be completely crystalline, it was assumed that it needed to reach its critical supersaturation ratio of ~3.5 on the surface [10] while still having ample time to undergo crystal growth before the glass formers reached their own critical concentrations for phase separation. It was also assumed that leucine would be partially amorphous if the amount of time it took to reach its critical concentration was only marginally smaller than that for the other components so that their glass formation would interfere with its crystallization and growth. Finally, it was assumed that the leucine content would be completely amorphous if other components reached their critical concentration for phase separation well before leucine had had an opportunity to nucleate instantaneously. The critical concentration of trehalose was assumed to be 830 mg/mL [10]. The exact value of the bevacizumab’s solubility was unknown. A critical concentration of 100 mg/mL was assumed, based on the observation of a sharp increase in solution viscosity at around 50 mg/mL during solution handling. For simplicity, co-amorphization of trehalose and bevacizumab was not considered.

##### Moxidectin–Leucine System in Water and Ethanol

Moxidectin is a small-molecule active that is insoluble in water and freely soluble in ethanol. It was found to reliably spray dry into an amorphous form during preliminary tests. Due to the solubility limitations involved, a water–ethanol cosolvent system was selected to solubilize both components. A screen on moxidectin at increasing ethanol mass fractions of 0.25, 0.5, and 0.75 resulted in increasing solubility values of 0.4, 10, and greater than 60 mg/mL, respectively. Leucine showed the opposite trend, losing solubility quickly with increasing ethanol mass fraction in the cosolvent [30]. Based on these data, a 1:1 *w*/*w* water–ethanol cosolvent system was chosen to allow for sufficient leucine solubility, i.e., approximately 5 mg/mL. It has previously been shown that the ethanol mass fraction in multi-solvent droplets at this initial solvent ratio decreases rapidly as evaporation progresses [22]. This fact rendered exact particle formation modeling impossible, as the critical concentration of each component changes over time with varying solvent composition in this situation, and such concentration profiles were not available for this system. However, the general trends predicted by the model could still be used to design the formulations. To allow sufficient time for leucine crystallization, the formulation intended to produce crystalline leucine was prepared near leucine saturation to enable it to reach critical saturation at the earliest possible time before the moxidectin. In the formulation intended to produce the least crystalline leucine, the time for reaching critical concentration was delayed relative to moxidectin by choosing a low initial saturation (5%). This approach favors early phase separation of moxidectin relative to leucine, because in this case, the solubility of leucine increases upon evaporation while that of moxidectin decreases due to the changing solvent environment.

##### Mannitol–Leucine System in Water

Both mannitol and leucine can crystallize during spray drying; hence, controlling their sequence of nucleation was the objective of the formulation design. Particle formation models were used to choose three formulations, such that mannitol would nucleate first, both leucine and mannitol would nucleate approximately at the same time, and, finally, leucine would nucleate before mannitol. As before, a critical supersaturation ratio of 3.5 resulting in a critical concentration of ~77 mg/mL was selected for leucine. For mannitol, the critical supersaturation was calculated according to the methodology proposed by He et al. [28], assuming a solubility of 216 mg/mL, a crystal density of 1520 kg/m^3^, and a water activity coefficient of ~1. These parameters resulted in a critical supersaturation of ~1.6 for mannitol in water, corresponding to a critical concentration of ~337 mg/mL. Using these concentrations for a constant total feed concentration of 40 mg/mL, the times for onset of nucleation for the mannitol and leucine at different fractions were calculated to select the formulations, based on the objectives described above.

The selected formulations for all three model systems and their predicted solid phases are presented in Table 1. The data in the column labeled *t*_w,leu_ refer to the predicted time difference between the two points in time during droplet evaporation, at which the active and leucine reached their respective critical concentrations. A negative value indicates that the active (or trehalose if it phase-separated earlier) reached critical concentration before leucine and likely initiated phase separation first.

#### 2.2.2. Spray Drying

Spray drying was performed on a custom lab-scale spray dryer with a nominal gas flow rate of 35 kg/h. Spray solutions were pumped into a pre-heated spray dryer via a peristaltic pump. The liquid streams were then atomized using a two-fluid nozzle (Model ¼ J, 1650 liquid body and 64 air cap, Spraying Systems Co., Wheaton, IL, USA), and the spray-dried particles were collected using a 5 cm cyclone separator.

For the bevacizumab model system, as-received bevacizumab solution was dialyzed into 1mM phosphate buffer using a dialysis membrane with a molecular mass cutoff of 10 kDa, and then diluted with trehalose and L-leucine solutions to achieve the desired formulation ratios and a total feed concentration of 40 mg/mL. The spray drying outlet temperature was 50 °C, the atomization pressure was 241 kPa, the liquid feed rate was 5 g/min, and the inlet temperature was 95–105 °C.

For the mannitol model system, mannitol and L-leucine were dissolved in deionized water to achieve the desired formulation ratios with a total concentration of 40 mg/mL. The spray drying outlet temperature was 50 °C, the atomization pressure was 138 kPa, the liquid feed rate was 10 g/min, and the inlet temperature was 120–130 °C.

For the moxidectin model system, moxidectin and L-leucine in different ratios were dissolved in a mixture of ethanol and water at a 1:1 mass ratio. Feeds with total solids concentrations of 5 and 10 mg/mL were spray-dried for the 95/5 formulation and the 70/30 and 50/50 formulations, respectively. The spray drying outlet temperature was 50 °C, the atomization pressure was 103–138 KPa, the liquid feed rate was 10 g/min, and the inlet temperature was 110–125 °C.

To approximate the initial atomized droplet size distribution, which is necessary for the particle formation models, an aqueous solution with 40 mg/mL of trehalose was spray-dried with the process conditions used for the drying of the bevacizumab system. Analysis of the primary particle size of the collected powder using an aerodynamic particle sizer in conjunction with a powder disperser, as described below, revealed a mass median aerodynamic diameter of 3.2 µm. Using a mass balance equation [4] and a particle density equal to 1580 mg/mL for amorphous trehalose [10], the mass median diameter of the atomized spray droplets was then back-calculated to be equal to about 9 µm. Because of the relatively high manufacturing yield of 83%, it was assumed that this initial median droplet size approximated the totality of the spray droplets. This result was used for the mannitol formulation calculations as well, although the process conditions were slightly different.

#### 2.2.3. Raman Spectroscopy

The solid state of the individual components of the spray-dried samples was analyzed using an in-house dispersive Raman spectrometer [31]. The raw crystalline materials of L-leucine and trehalose dihydrate were measured, with the results providing the reference spectra for the most stable crystalline polymorphs of these compounds, α polymorph for leucine and β polymorph for mannitol. However, it was later found that the residual spectra for some of the leucine-containing formulations could not be minimized when the α-leucine reference spectrum was used for the spectra deconvolution. Another conformational polymorph of leucine was previously observed at temperatures higher than 80 °C [32]. It was believed that such a less stable phase of leucine also appeared in spray-dried particles. In this study, the Raman spectra of a less stable polymorph of leucine, β-polymorph, was obtained from the residual spectra of spray-dried bevacizumab–trehalose–leucine formulation (B10T40L50) after subtracting the spectral contribution of bevacizumab and trehalose. The obtained reference spectrum manifested different features compared to that of α-leucine and was confirmed to be for the β polymorph of leucine [32].

A spray-dried batch of trehalose–bevacizumab with a mass ratio of 4:1 (consistent with the leucine-containing spray-dried formulations) was sampled to provide the reference spectrum for this specific amorphous mixture. Spray-dried trehalose was also used as the amorphous reference spectrum [10]. The previously determined spectra of different polymorphs of mannitol were used to confirm its crystallinity [33].

The amorphous reference spectrum for leucine was obtained by subtracting the amorphous trehalose reference from the spectrum obtained from the spray-dried trehalose–leucine powder collected at the exhaust of the cyclone. To this end, another custom lab-scale spray dryer [34] was used to spray dry a specific formulation of trehalose and leucine in order to measure the Raman spectra of amorphous leucine. The production of completely amorphous leucine particles with no crystalline signal within the detection limit of the Raman spectroscopy has not been accomplished; hence, both the formulation and process conditions were fine-tuned to minimize the nucleation and crystal growth of leucine. A low mass fraction of 10% of leucine was selected. A relatively large air-to-liquid ratio was further chosen to decrease the initial atomized droplet size [35], and the inlet gas temperature was set to a relatively high value. These process conditions minimized the evaporation time for the droplets and hence, decreased the time available for crystallization of leucine [5]. Furthermore, operating on the assumption that the small particles dried from the smallest of the atomized droplets have a higher possibility of being completely amorphous [10], an in-line filter was used to collect particles downstream of the cyclone. Considering the low cut-off values of the cyclone, most of the particles not collected were assumed to be sub-micron sized. These collected sub-micron particles were then used to obtain the reference spectrum for amorphous leucine, as reported previously [36].

**Figure 1:**
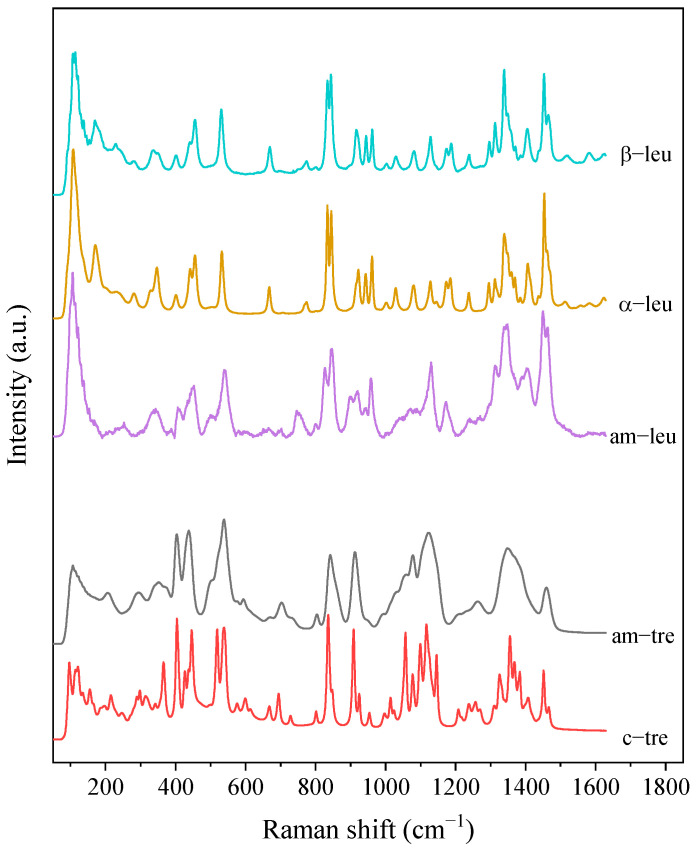
The measured reference Raman spectra of different solid phases for leucine (crystalline alpha = α-leu, crystalline beta = β-leu, and amorphous = am-leu) and trehalose (amorphous = am-tre, crystalline trehalose dihydrate = c-tre).

The obtained reference spectra for different phases of leucine and trehalose are shown in Figure 1. The reference spectra for the 1:4 *w*/*w* bevacizumab–trehalose mixture and the amorphous moxidectin are presented in the corresponding figures in the results and discussion section.

#### 2.2.4. Powder X-ray Diffraction (PXRD)

Powder X-ray diffraction was used to identify the solid phases of L-leucine and mannitol, where applicable, in the formulations. Diffractograms were collected on a diffractometer (MiniFlex 600, Rigaku Corporation, Tokyo, Japan) using a copper anode generator set to 45 kV and 15 mA. Powder samples were gently compressed onto a zero-background sample cup and scanned over a range of 3–40° 2θ at 2.5°/min with sample rotation.

#### 2.2.5. Differential Scanning Calorimetry (DSC)

Thermal analysis of samples was performed using a temperature-modulated differential scanning calorimeter (DSC 3+, Mettler Toledo, Columbus, OH, USA). To quantify the “dry” properties of samples, 2–6 mg of material was hermetically sealed in a 40-µL aluminum pan using Mettler Toledo piercing lids. Immediately prior to analysis, the instrument vented the pan to enable moisture to evaporate during heating. For quantification of the “wet” glass transition temperature, samples were hermetically sealed in pans that were not vented, to maintain the residual water content during analysis. For the mannitol formulations, samples were scanned from 25 to 200 °C at a rate of 10 °C/min. For the bevacizumab formulations, samples were scanned with temperature modulation from 0 to 160 °C at a rate of 2.5 °C/min and a modulation amplitude of 1.5 °C per 60 s period. Similarly, for the moxidectin formulations, samples were scanned from 25 to 150 °C at a rate of 2.5 °C/min and a modulation amplitude of 1.5 °C per 60 s period.

#### 2.2.6. Karl Fischer (KF) Titration

The water content of spray-dried powders was quantified by Karl Fischer titration using a coulometric oven titrator (Metrohm 851 Titrando KF, Metrohm USA Inc., Tampa, FL, USA). Sample masses from 10 to 50 mg were hermetically sealed in a crimped KF vial and heated to an oven temperature of 105 °C prior to analysis. A diaphragm-less mode was used for the generator electrode.

#### 2.2.7. Ultramicroscopy

Electron micrographs were obtained using a field-emission scanning electron microscope (Sigma 300 VP, Zeiss, Jena, Germany) operating at an accelerating voltage of 5 kV and a working distance of 10–14 mm. Minute quantities of the powder samples were placed on double-sided adhesive carbon tapes attached to ordinary aluminum sample stubs. To minimize the interactions between the electron beam and the organic particles, the samples were first coated with a layer of gold nanoparticles to a thickness of ~10 nm using a vacuum sputter coater (Desk II, Denton Vacuum LLC, Moorestown, NJ, USA). The morphology of all the spray-dried formulations at each time point and storage conditions was assessed from the obtained micrographs, with special attention given to detecting any morphological changes such as interparticle fusion or the appearance of fibers.

To capture higher-resolution micrographs with minimal beam damage, and to assess the morphology of fibers without the presence of any coating, helium ion microscopy (ORION, Zeiss, Jena, Germany) was performed on a limited number of samples using an accelerating voltage of 29 kV, a working distance of ~9 mm, and an aperture of 10 µm. About five micrographs were taken at each time point and for each sample, to qualitatively assess the number and frequency of appearances of fibers. If the fibers were approximately longer than ¼th of the median particle diameter, they were counted as long fibers, with the rest counted as short fibers.

#### 2.2.8. Aerodynamic Particle Size Distribution (APS)

The aerodynamic particle size distribution of the spray-dried powders was measured using an aerodynamic particle sizer with aerosol diluter and small-scale powder disperser (Models 3321, 3302 and 3433, TSI, Shoreview, MN, USA). In the diluter, a 100:1 capillary setting was selected at a pressure of 80 Pa. The powder disperser air-flow and sheath-flow rates were set at 18.5 and 4 L/min, respectively. Because of the strong dispersion forces exerted on the samples in the powder disperser, the size distribution obtained using the APS was assumed to be representative of the primary particle size. Each particle size distribution was measured for 20 s and recorded in triplicates. The data were then processed assuming a log-normal distribution to determine the mass median aerodynamic diameter (MMAD) and the geometric standard deviation (GSD) of the primary particle size distribution.

#### 2.2.9. Aerosol Performance Characterization Using a Next Generation Impactor (NGI)

The aerosol performance was characterized using a next generation impactor (MSP NGI Model 170, MSP Corp., Shoreview, MN, USA) with a high-resistance dry powder inhaler (Plastiape S.p.a., Osnago, Italy). A total of 10 mg of dry powder was hand-filled into size 3 DPI-grade capsules (Vcaps Plus, Capsugel, Morristown, NJ, USA). NGI stages were pre-coated with a 1% solution of Tween 20 in ethanol. A total of 2 mL of solution was evenly distributed across each large stage (1 and 8), and 1 mL across each small stage (2–7). Pans were allowed to dry fully before use (at least 2 h). A pump provided flow through the DPI for 4 s at 65 L/min. A pre-separator containing 10 mL of diluent was used upstream of the NGI. After a single actuation, the NGI pans, device, induction port, and pre-separator were rinsed with a known quantity of the diluent and the rinsate assayed for drug concentration.

Using the obtained concentrations together with the knowledge of the cut-off diameters of each stage, the mass median aerodynamic diameter was obtained by assuming a log-normal distribution [1]. The emitted dose was obtained as the mass fraction of the loaded dose that left the device. The fine particle dose was defined as the mass fraction of the loaded powders collected in the impactor with aerodynamic diameters of less than 5 microns.

#### 2.2.10. Active Concentration

The active concentration in the bevacizumab-containing powders was quantified by UV fiber optic probes (Rainbow MicroDISS Profiler™, Pion, Billerica, MA, USA, 20 mm path length). A total of 6–10 mg of powder was dissolved in 5 mL of pH 7.4 phosphate-buffered saline. The second derivative of the absorbance spectra was quantified over the range of 280–284 nm and compared with a standard curve ranging from 0 to 300 µg/mL. The measured concentration was reported on a dry basis and was corrected for water content.

The active concentration of the mannitol-containing powders was quantified by HPLC (Model 1100, Agilent, Santa Clara, CA, USA). A total of 4–8 mg of powder was dissolved in 5 mL of 50/50 *v*/*v* acetonitrile/water. An isocratic method was used with a mobile phase of 70/30 acetonitrile/water at a flow rate of 2.2 mL/min and 20 µL injection volume. The column (TSKgel Amide-80 3µm, 4.6 × 150 mm, Tosoh Bioscience, South San Francisco, CA, USA) was heated to 70 °C. Absorbance was quantified at 200 nm against a standard curve ranging from 0 to 1100 µg/mL.

The active concentration of the moxidectin-containing actives was quantified by HPLC (Model 1100, Agilent, Santa Clara, CA, USA). A total of 3–6 mg of powder was dissolved in 10mL of 90/10 *v*/*v* methanol/water. A 2 min isocratic method was used with a mobile phase of 90/10 *v*/*v* methanol/water at a flow rate of 1.5 mL/min and 5 µL injection volume. The column (Agilent Poroshell 130 EC-C18 2.7um, 4.6 × 50 mm) was heated to 30 °C. Absorbance was quantified at 242 nm against a standard curve ranging from 0 to 250 µg/mL.

#### 2.2.11. Stability Analysis

The solid-state, morphology, aerosol performance, and thermal properties of all spray-dried formulations were screened at two different stress conditions of 25 and 40 °C for up to 8 months. The time points and the characterizations performed for the three model systems are shown in Table 2.

The detailed methodology used for storing the solid powders to minimize moisture exposure has been explained previously [37]. Briefly, necessary quantities of each formulation, intended for each time point and stress condition, were placed in 3.7 mL glass vials (Cat. No. 03-338A, Fisher Scientific, Ottawa, ON, Canada) and capped, while being purged by dry air. Each vial was then placed in an aluminum bag (Prod. No. 139-312, Ted Pella Inc., Redding, CA, USA) in conjunction with a desiccant pouch equilibrated at 10% RH to prevent over-drying of the powders. This bag was then double heat-sealed and put into another aluminum bag with another desiccant pouch equilibrated at 0% RH. Each external aluminum bag was also double sealed. PXRD, NGI, APS, KF, and DSC measurements were only conducted at two time points, as the amount of powder required for these measurements was much more than the amount required for SEM and Raman spectroscopy.

## 3. Results and Discussion

To interrogate the correlation between the solid phase of L-leucine in the spray-dried powders and their storage stability, formulations were designed to target varying levels of amorphous leucine. The spray-dried powders were characterized and studied at t_0_ and the subsequent time points. As explained before, three model systems were chosen:

1. A large amorphous API in an amorphous excipient matrix (bevacizumab and trehalose);

2. A small-molecule amorphous API (moxidectin);

3. A crystallizing excipient (mannitol), which can also stand in for APIs that crystallize during spray drying.

These systems cover a broad range of formulation design applications, suggesting the general trends for other leucine-containing powders. As discussed in detail in the methods section, three formulations of each model system were designed to target low, moderate, and high fractions of leucine crystallinity.

### 3.1. Particle Morphology

The morphology of the spray-dried particles and their evolution during the stability challenge is discussed first.

**Figure 2:**
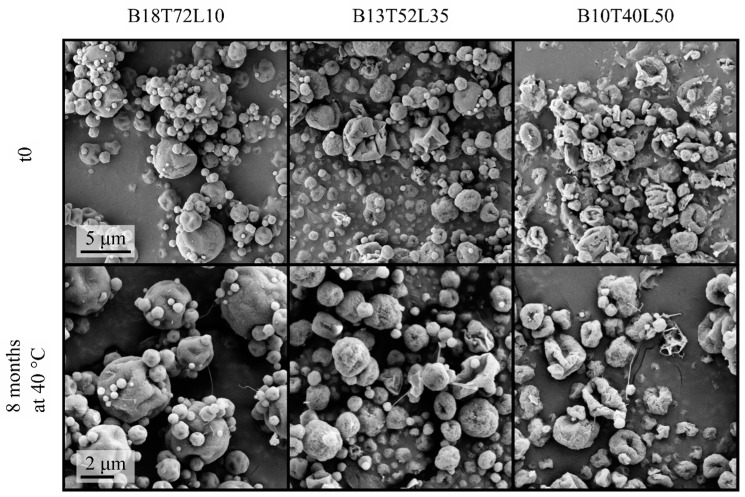
Electron micrographs of the spray-dried bevacizumab–trehalose–leucine formulations at t_0_ and after 8 months of storage at 40 °C. Note the appearance of a small number of thin fibers in the bottom row. The scale bars apply to all micrographs in each row.

The spray-dried bevacizumab formulations at t_0_ are shown in the first row of Figure 2. As expected, at higher leucine mass fractions, the particles were generally more wrinkled and appeared to have lower particle densities, all of which are characteristics of particles with a high crystalline leucine content. The morphologies of these formulations stored at 25 and 40 °C were monitored at different intervals with no major morphological changes observed even after 8 months of storage at 40 °C, as shown in Figure 2.The particles retained their shape and surface morphology, and no fusing was observed, indicating excellent physical stability. However, as seen in the bottom row of Figure 2, thin fibers appeared in these formulations. It was observed that the number and frequency of the appearances of these fibers increased with the storage temperature and decreased with the increasing leucine fraction in the formulation; i.e., the formulations with the smallest overall amount of leucine had the most fibers. This fact points to the possibility of a physical transformation during the storage of leucine in its unstable states, because the formulation with small amounts of leucine was designed to have a large amorphous leucine fraction. The protein was not expected to be able to form such fibers, and fiber formation was not seen in other trehalose-containing formulations without leucine [38]. Furthermore, the number of fibers was seen to increase with storage time, ruling out any possible role of foreign particulate matter.

**Figure 3:**
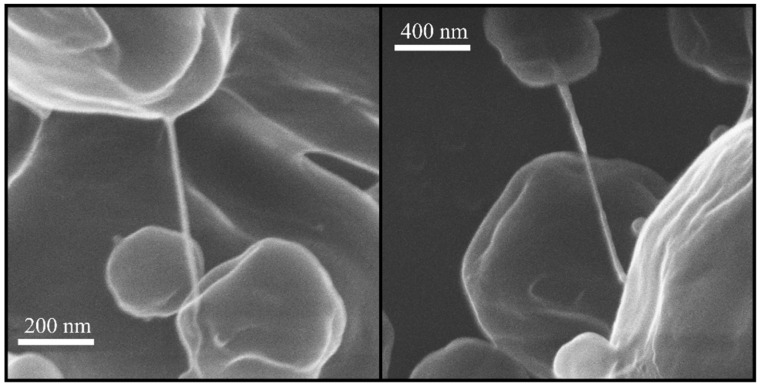
Helium ion micrographs of some of the fibers in the B18T72L10 formulation after 1 month of storage at 40 °C.

A helium ion microscope was used to image the fibers at higher magnifications in order to minimize the beam damage and preclude the possibility of the coating masking their actual shape. Two fibers from the B18T72L10 formulation after one month of storage at 40 °C are shown in Figure 3. The fibers had a high aspect ratio with lengths reaching several hundred nanometers and they were very thin with thicknesses approximately between 10 and 20 nm.

**Figure 4:**
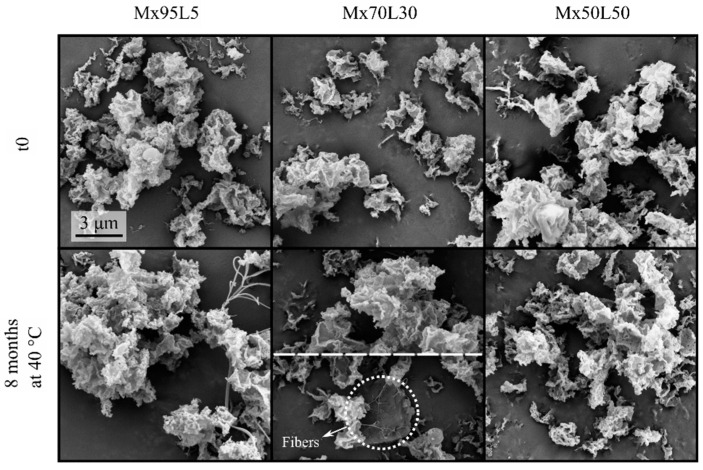
Electron micrographs of the spray-dried moxidectin–leucine formulations at t_0_ and after 8 months of storage at 40 °C. Some fibers can be seen in the left panel of the bottom row. Only a few fibers could be seen for the Mx70L30 after 8 months of storage. For this case, the top half shows a typical field of view without fibers. The bottom half shows small fibers that were occasionally seen (dotted circle). No fibers can be seen in right panel of the bottom row. The scale bar applies to all micrographs.

The spray-dried powders containing moxidectin and leucine are shown in Figure 4. In all formulations, the particles were thin-shelled and appeared to have low particle densities, probably due to the very low amorphous solubility of moxidectin in the cosolvent system, which in turn, could cause early phase separation during droplet evaporation. For up to 8 months of the stability challenge, no significant morphological change was observed. Particles retained their appearance without any signs of fusing. As in the first model system, fibers started appearing in the formulation with the least amount of leucine (Mx95L5) after 2 months of storage at 40 °C. After 8 months of storage at 40 and 25 °C, the Mx95L5 samples had developed many long fibers and some shorter ones. Only after 8 months were a very small number of fibers detected for the Mx70L30 formulation stored at the higher temperature of 40 °C. No fibers were detected for the Mx50L50 formulation.

**Figure 5:**
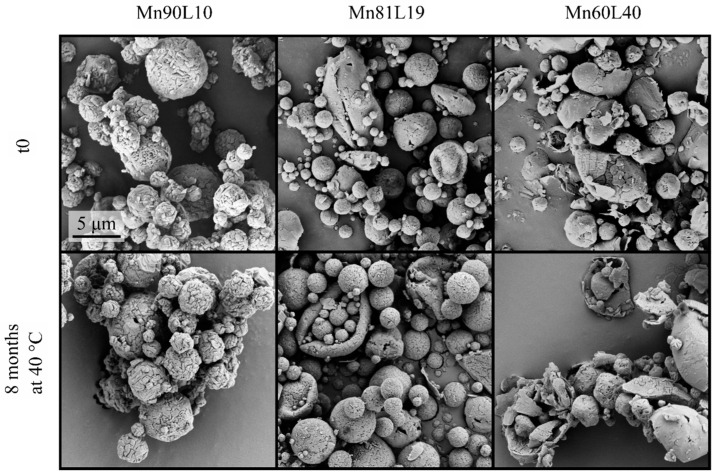
Micrographs of the spray-dried mannitol–leucine formulations at t_0_ and after 8 months of storage at 40 °C. The scale bar applies to all micrographs.

The morphology of the mannitol-containing powders is shown in Figure 5. The particles with the highest mannitol content had discernible nanoparticles with a regular morphology on the surface and a spherical, hollow structure. This formulation was designed to allow mannitol to crystallize earlier than leucine. The resulting morphology is consistent with mannitol crystals on the surface [39]. At higher leucine contents, where the crystallization sequence was predicted to be reversed, the larger particles had collapsed and were hollow with a thin shell, which is consistent with typical leucine particle morphology [18]. Many of the particles with the highest leucine fraction (Mn60L40) were broken, probably during the cyclone separation. As seen from the bottom row of this figure, all formulations were morphologically stable. No fibers were detected for any of the powders at any time point or in any storage condition.

### 3.2. Solid State and Chemical Composition

#### 3.2.1. Bevacizumab

**Figure 6:**
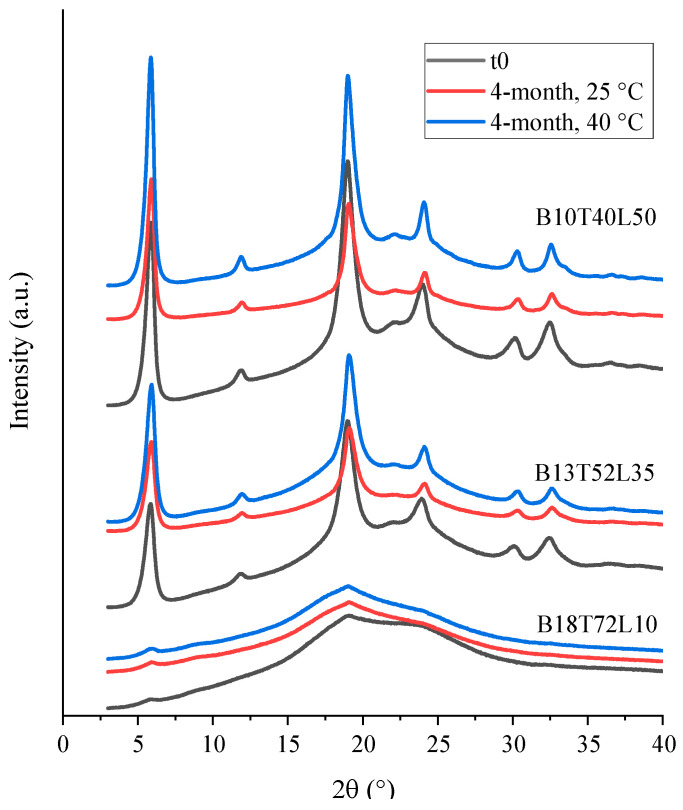
PXRD diffractograms of the spray-dried bevacizumab–trehalose–leucine powders at t_0_ and after 4 months of storage at 25 and 40 °C.

The solid state of the formulations was assessed using both the PXRD and Raman spectroscopy. The PXRD diffractograms of the bevacizumab powders at t_0_ and the 4-month time point are shown in Figure 6. Based on these diffractograms, the powder with the least amount of leucine (B18T72L10) was mostly amorphous, consistent with the modeling predictions, while the other two formulations with more leucine (B13T52L35 and B10T40L50) were partially crystalline. No significant change in the diffraction patterns of the powders was observed between the freshly dried powders and those stored for four months at 25 and 40 °C. The assay did not detect any crystallinity in the bevacizumab–leucine powder that showed the most fibers (B18T72L10), indicating that any possible re-crystallization during fiber formation affected a portion of leucine that was below the limit of detection for PXRD.

**Figure 7:**
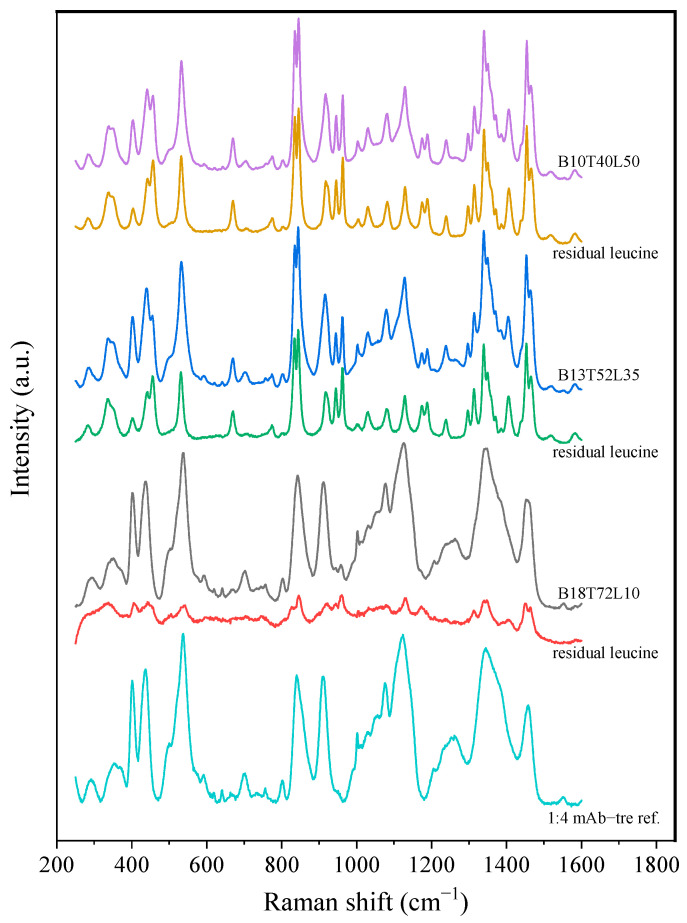
The raw and residual leucine spectra of the bevacizumab formulations after 8 months of storage at 40 °C. No significant difference was detected between the t_0_ and 8-month measurements.

The Raman spectra of these powders were measured and deconvoluted for each time point up to 8 months after production, with no change observed during storage. To determine the solid phase of the leucine, the reference spectrum for the 1:4 *w*/*w* bevacizumab–trehalose mixture was subtracted from the raw spectra to result in the residual leucine spectra shown in Figure 7 for the powders after 8 months of storage at 40 °C. The residual spectra were then compared to the reference spectra shown in Figure 1. Based on these measurements, the first formulation with only 10% leucine (B18T72L10) was completely amorphous, as expected. The bevacizumab and trehalose contents in the other two formulations (B13T52L35 and B10T40L50) were amorphous, while leucine was completely crystalline within the detection limits of the method. However, the measured spectra resembled those of the β form, which is the less stable leucine polymorph. As shown in Table 3 the deconvolution of the Raman spectra confirmed the stability in the solid phase of these powders. Any possible changes due to fiber formation remained below the limit of detection.

The glass transition temperature, *T*_g_, of the powders was measured using DSC to confirm that the protein–trehalose mixed amorphous phase had not separated during storage. In general, for the amorphous systems, a higher *T*_g_ translates to a more physically stable product with a longer shelf-life, because a high *T*_g_ ensures low molecular mobility at typical storage temperatures. As presented in Table 4, the measured dry *T*_g_ of the formulations containing bevacizumab, trehalose, and leucine remained unchanged during storage and were all in the range of 118 to 124 °C. The dry *T*_g_ was used to determine if phase separation of the protein and sugar occurred during storage. The initial wet *T*_g_ of the particles was between 49 and 52 °C (not shown) due to the plasticizing effect of the residual moisture. Despite the proximity of the wet *T*_g_ to the 40 °C storage condition, the material was stable. The measured glass transition temperature in this case represents the average across the particles. However, it has been shown that solid phase properties, including the glass transition temperature, in core–shell type particles are a function of the radial position [40]. The particles tested here either had a crystalline hydrophobic shell or were enriched on the surface with the high *T*_g_ protein, which was expected to provide additional protection. The employed methodologies for dry storage successfully prevented further moisture uptake. In fact, upon storage, all powders lost some of their water content.

The concentration of bevacizumab is also shown in Table 4. For all cases, the concentration was close to the target value and did not decrease significantly with time, demonstrating the chemical stability of bevacizumab regardless of the appearance of the fibers. Overall, the solid state and chemical composition of the bevacizumab powders remained constant during storage.

#### 3.2.2. Moxidectin

**Figure 8:**
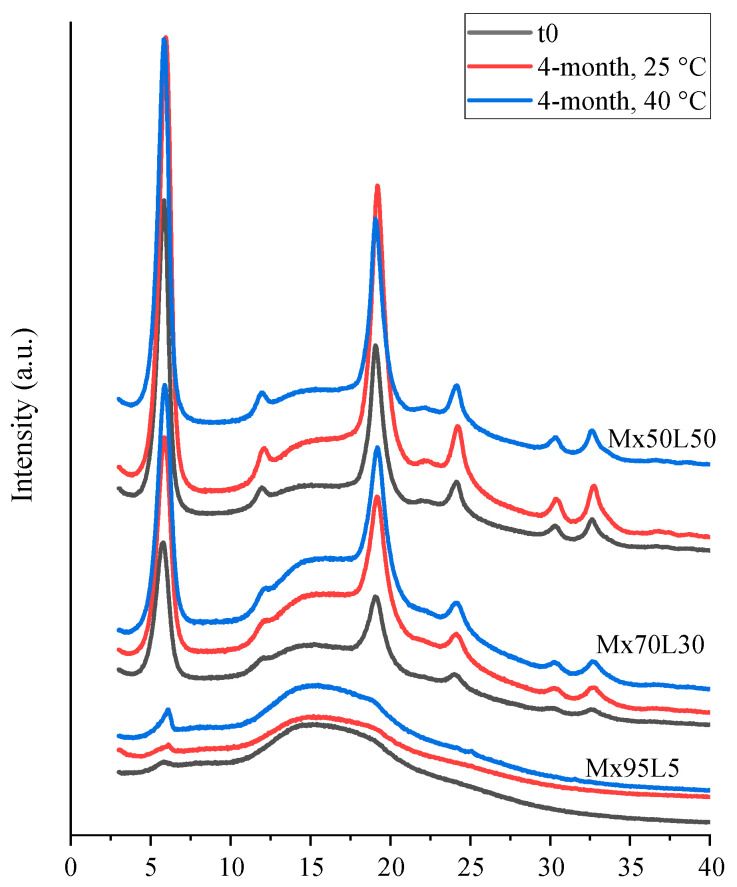
PXRD diffractograms of the spray-dried moxidectin–leucine powders at t_0_ and after 4 months of storage at 25 and 40 °C.

**Figure 9:**
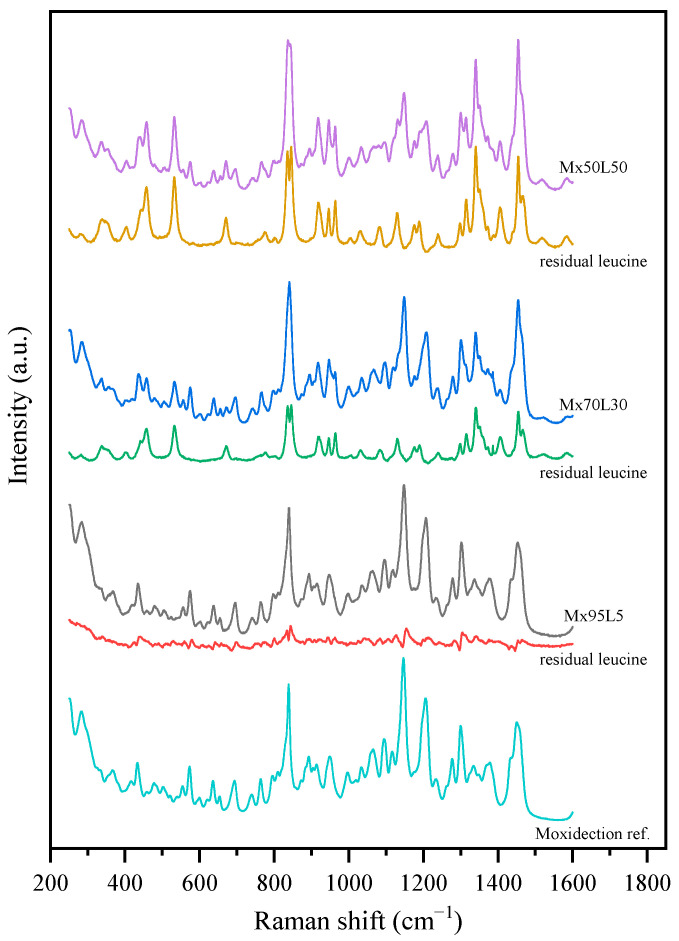
The raw and residual leucine spectra of the moxidectin formulations after 8 months of storage at 40 °C. No significant difference was detected between the t_0_ and 8-month measurements.

The PXRD diffractograms of the moxidectin–leucine powders are shown in Figure 8. Obvious crystalline peaks attributable to leucine could be observed for the powders containing 30% and 50% leucine, while the powder containing only 5% leucine showed weak peaks with a stronger amorphous background. Some peaks appeared during storage for the 5% leucine formulation. This could be attributed to the detection limit of the PXRD measurements and the dependence on the crystal size as Raman measurements confirmed that leucine was completely crystalline in this formulation. Raman spectroscopy could not detect amorphous leucine in any of the formulations, while moxidectin was completely amorphous. The raw and residual leucine spectra of these formulations are shown in Figure 9, only for the powders stored for 8 months at 40 °C, as there was no significant change in the spectra over time. As shown in Table 5, it was determined that the crystalline leucine in the Mx70L30 and Mx50L50 formulations resembled the β-form, i.e., the less stable polymorph. The residual spectra obtained from the Mx95L5 were of relatively poor quality, providing only confirmation that the majority of the leucine was in crystalline form; however, its exact polymorphism could not be determined. In summary, neither the PXRD nor Raman measurements revealed any significant changes in the solid state after 8 months of storage at 25 and 40 °C.

The dry glass transition temperatures of the moxidectin formulations as measured by DSC are shown in Table 6. In each formulation, the initial $T_{g}$ ranged from 115 to 118 °C, indicating that the composition of the amorphous phase in all three systems was approximately the same. This result was expected, as only negligible amorphous leucine was mixed with the amorphous moxidectin domain. No significant changes in the $T_{g}$ were observed during the stability study.

Additionally, as seen in Table 6, the initial water content of the moxidectin formulations was low even in the amorphous state, ranging from 0.49 to 0.66%, owing to the low hygroscopicity of moxidectin (logP = 5.4) [41]. After 4 months of stability hold, the water content decreased slightly, ranging from 0.35 to 0.46%, due to the presence of desiccant in the packaging.

As seen in Table 6, the moxidectin concentration within each formulation was close to the expected value and did not change during the stability study, indicating good chemical stability under these conditions.

#### 3.2.3. Mannitol

**Figure 10:**
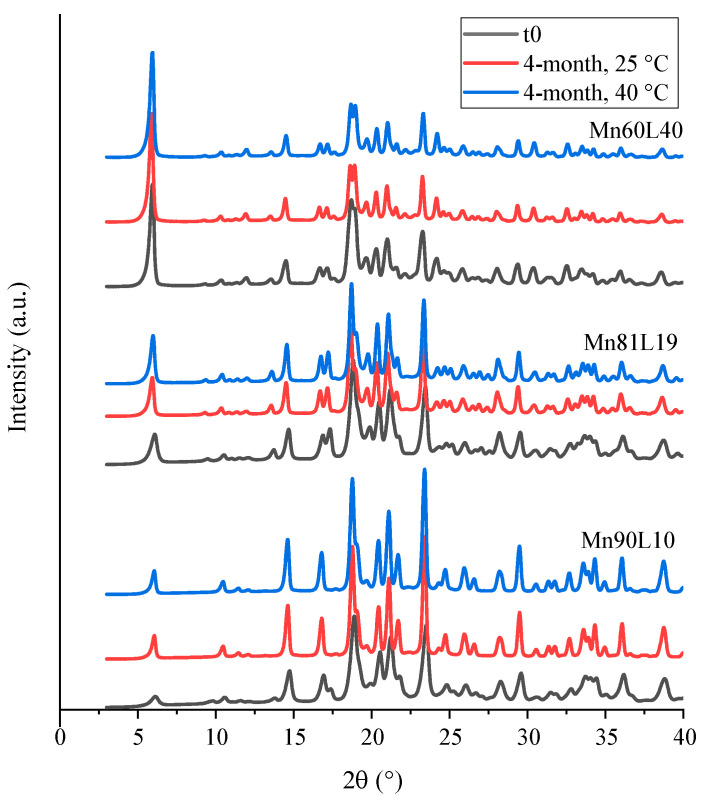
PXRD diffractograms of the spray-dried mannitol–leucine powders at t_0_ and after 4 months of storage at 25 and 40 °C.

The diffractograms of the mannitol–leucine powders are shown in Figure 10. No amorphous halo was observed, demonstrating that all mannitol-containing powders were highly crystalline. The PXRD diffractograms of the powders showed some variations in the peak patterns during storage, especially in the powders with a higher mannitol content (Mn90L10 and Mn81L19). This variability was likely due to the interconversion of different mannitol polymorphs [33]. Raman spectroscopy (not shown) confirmed that leucine was in the most stable crystalline α-form, both initially and during storage. No amorphous leucine was detected by the Raman spectroscopy.

The water content of the mannitol formulations presented in Table 7 was less than 0.1% in all cases, a value consistent with a fully crystalline phase for all components in all formulations. No significant changes were observed during the stability challenge.

As expected for a crystalline powder, the DSC traces for these formulations did not show a glass transition. The melting point of the mannitol in the formulations as measured by DSC is also shown in Table 7. All three had similar onsets of melting at ~164 °C as anticipated for crystalline mannitol [39], and no significant changes were observed during the stability challenge.

As shown in Table 7, the mannitol concentration was approximately equal to the expected values. No change could be observed after storage, pointing to excellent chemical stability in the presence of crystalline leucine.

### 3.3. Aerosol Properties

#### 3.3.1. Bevacizumab

**Figure 11:**
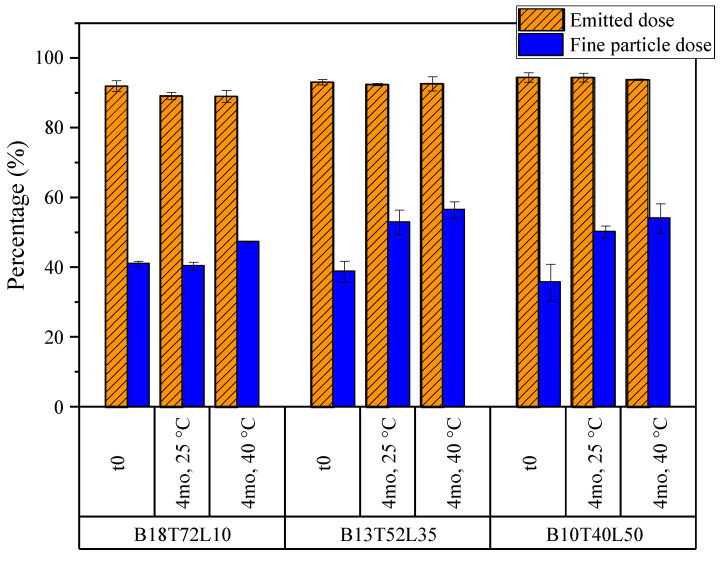
The emitted doses and fine particle doses of the bevacizumab formulations obtained from NGI measurements. The fine particle dose is defined as the fraction of the emitted particles with aerodynamic diameters of less than 5 microns. The error bars correspond to one standard deviation.

The aerosol properties of the formulations were compared before and after 4 months of stability holds at 25 and 40 °C. These properties were assessed via the aerodynamic particle size (APS) measurements of the powders forcefully dispersed with a small-scale powder disperser, and also via a next-generation impaction (NGI) measurement of the aerosol emitted from a capsule-based dry powder inhaler. The emitted dose, defined as the fraction of the mass of the powder that left the device relative to the total filled dose, and the fine particle dose (FPD), defined as the mass fraction of the emitted dose that was collected in the impactor with aerodynamic diameters less than 5 microns, were obtained from the NGI measurements and are shown in Figure 11 for the bevacizumab system. The emitted dose of these formulations remained high at all time points, at around 90%. No decrease in the fine particle dose was observed in storage for any formulation. In fact, the FPD tended to increase slightly, perhaps due to the decreased water content (as seen in Table 4).

**Figure 12:**
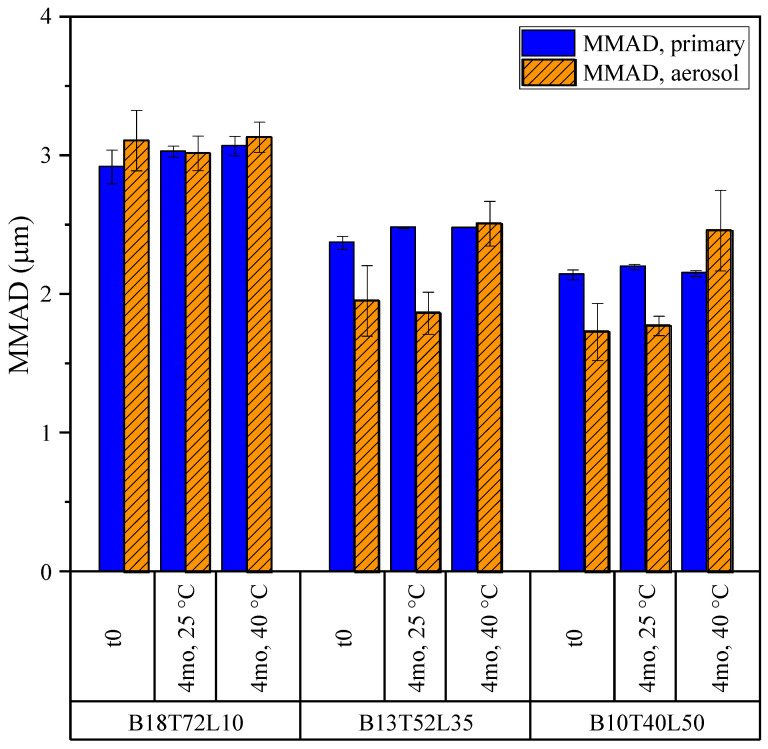
The mass median aerodynamic diameters (MMADs) of the bevacizumab formulations for the primary particle size and aerosol size distribution from a DPI. The error bars correspond to one standard deviation.

The mass median aerodynamic diameters (MMAD) of the bevacizumab formulations obtained by the APS and NGI measurements are shown in Figure 12. From the strong dispersion forces in the small-scale disperser, it can be assumed that the values obtained from the APS measurements approximate the size distribution of the primary particles, while the size distributions obtained from the NGI measurements describe the aerosol from an inhaler accounting for relevant cohesion and adhesion forces. It was observed that aerodynamically smaller particles were produced by increasing the leucine fraction. The primary particle size was in the respirable range for all powders and did not change on stability hold. The corresponding GSD was close to 1.6 for all formulations and conditions. No significant change in the aerosol MMAD was found during storage for the formulation with the least amount of leucine (B18T72L10). The MMADs for the other two formulations (B13T52L35 and B10T40L50) appeared to increase slightly for the powders stored at 40 °C. However, it is unclear whether these increases are statistically significant because the impactor measurements for these highly dispersible powders had appreciable interstage losses, which introduced additional variability in the stage-to-stage distribution. The fine particle doses in these cases were unaffected (see Figure 11).

Importantly, the B18T72L10 formulation, which formed the greatest number of fibers in storage, showed no adverse change in any of the particle size or aerosol performance metrics. Hence, it can be concluded that the occurrence of these fibers did not adversely affect the aerosol properties of the leucine-containing formulations.

#### 3.3.2. Moxidectin

**Figure 13:**
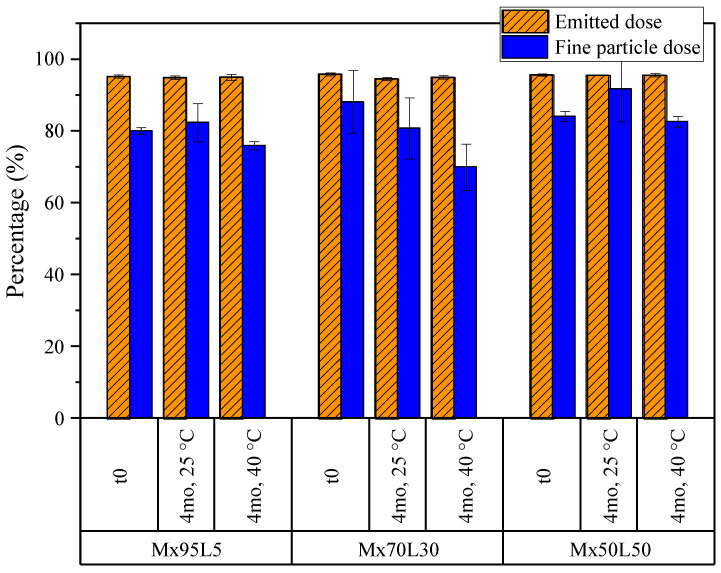
The emitted doses and fine particle doses of the moxidectin formulations obtained from NGI measurements. The fine particle dose is defined as the fraction of the emitted particles with aerodynamic diameters of less than 5 microns. The error bars correspond to one standard deviation.

As with the other formulations, the aerosol properties of the moxidectin formulations were compared before and after 4 months of stability holds at 25 and 40 °C. The emitted dose and fine particle dose obtained from the NGI measurements are shown in Figure 13. The formulations had very high emitted doses of about 95% in all conditions. The unusually high values of fine particle doses were due to the smaller particle sizes of these formulations upon spray drying out of a water–ethanol cosolvent system, which results in smaller atomized droplets [42]. No statistically significant decrease in the fine particle dose was detected, pointing to good stability of aerosol performance for all powders during storage.

**Figure 14:**
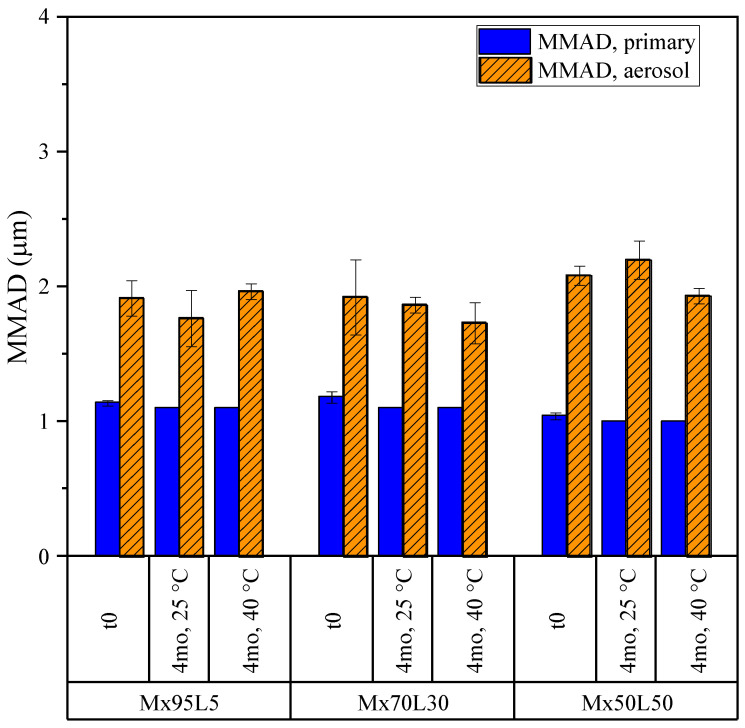
The mass median aerodynamic diameters (MMADs) of the moxidectin formulations for the primary particle size and aerosol size distribution from a DPI, obtained from APS and NGI measurements, respectively. The error bars correspond to one standard deviation.

The MMADs of the same powders obtained from the NGI and APS measurements are shown in Figure 14. The MMADs of the moxidectin formulations in storage remained close to 1 µm for the primary particles and close to 2 µm for the aerosol, resulting in a very high fine particle dose, as discussed above. The difference between the MMADs obtained for the primary particle size distribution and those obtained for the aerosol suggests is in the incomplete de-agglomeration of these powders in the inhaler. Regardless, no significant change in the aerodynamic properties of the powders was observed in storage.

The above observations yet again point to the conclusion that the appearance of the fibers in the moxidectin powders (mostly in the Mx95L5 formulation) did not adversely affect their aerosol performance.

#### 3.3.3. Mannitol

**Figure 15:**
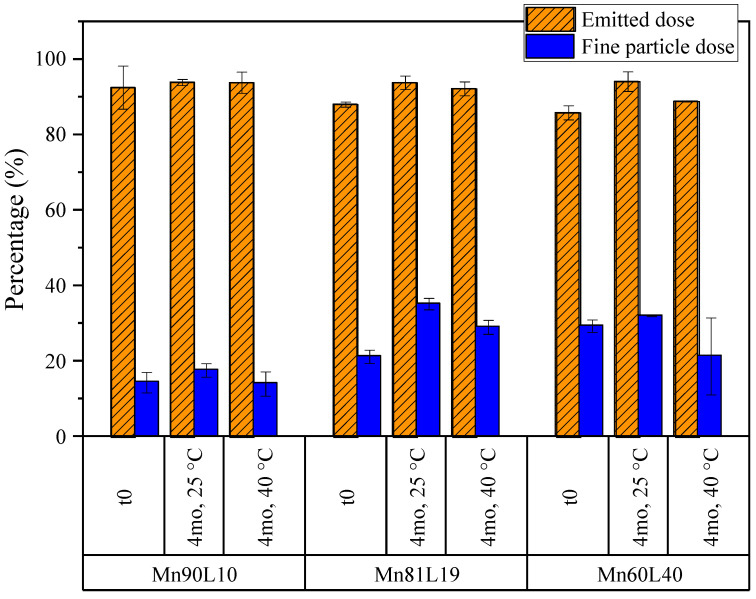
The emitted doses and fine particle doses of the mannitol formulations obtained from NGI measurements. The fine particle dose is defined as the fraction of the emitted particles with aerodynamic diameters of less than 5 µm. The error bars correspond to one standard deviation.

**Figure 16:**
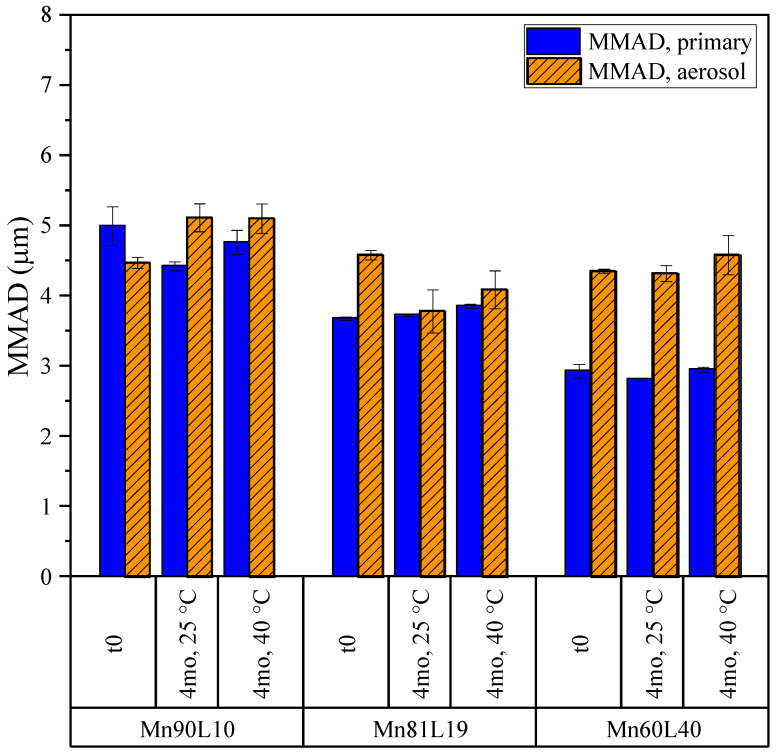
The mass median aerodynamic diameters (MMADs) of the mannitol formulations for the primary particle size and aerosol size distribution from a DPI, obtained from APS and NGI measurements, respectively. The error bars correspond to one standard deviation.

As with the previous systems, the aerosol properties of the mannitol–leucine powders were compared before and after 4 months of stability holds at 25 and 40 °C. The emitted doses and fine particle doses are shown in Figure 15. The emitted doses exceeded 80% for all formulations, but the fine particle doses were less than 30%, due to the much larger aerodynamic sizes of these particles. As seen in Figure 16, the MMADs of these powders obtained from NGI measurements were between 3 and 5 µm, resulting in the observed relatively small fine particle doses. Regardless, none of the tested particle or aerosol properties changed significantly during storage.

### 3.4. L-Leucine Solid State and Fiber Formation Kinetics

As demonstrated above, the bevacizumab, moxidectin, and mannitol formulations exhibited different tendencies to form fibers in storage. In this section, the correlation between leucine’s physical form and fiber growth is summarized. The study monitored the growth of fibers using scanning electron microscopy and the solid phase of leucine using the DSC, PXRD, and Raman spectroscopy at multiple time points (0.5 to 8 months). The appearance of fibers based on these observations is summarized in Table 8.

In the bevacizumab formulations, the L-leucine fibers appeared gradually and grew longer during the stability challenge. It was observed in all cases that fibers formed more slowly at 25 °C than at 40 °C. Fibers formed earlier for the 10% L-leucine formulation than for the others under both conditions. It was confirmed that this formulation, which was designed to allow strong interference of the protein with leucine crystallization, contained a detectable amorphous leucine phase. Amorphous leucine has a high dry glass transition temperature of 140 °C and thus, does not depress the glass transition of trehalose in leucine–trehalose mixtures [43]. However, the wet *T*_g_ measured for the trehalose–bevacizumab phase was significantly plasticized by the presence of water. This implies that the amorphous leucine present in the matrix may have sufficient mobility to recrystallize at the storage conditions in the study. Amorphous leucine has a higher energy state and is thermodynamically metastable compared with its crystalline forms. However, it is unknown whether small amounts of leucine are soluble within the amorphous trehalose/bevacizumab matrix, which could prevent phase separation and subsequent recrystallization of the leucine. Additionally, the two formulations in which amorphous leucine was not detected still formed fibers. However, as seen in Table 1 leucine was not present in its most stable polymorphic form, but rather in a form resembling the β polymorph, which is at a higher energy state than the most stable α form [32].

Despite the increasing fiber formation over the course of the stability challenge as observed in the electron micrographs, no measurable changes in the solid phase compositions were observed via the Raman spectroscopy for any formulation or at any of the time points. Given that the detection limit of the Raman technique for the components in a multi-component system is on the order of 0.1% mass fraction [44], this finding indicates that the bulk of the amorphous leucine phase did not re-crystallize.

As seen in Table 8, certain moxidectin formulations also exhibited the formation of leucine fibers during the stability challenge. For this system, fibers first appeared in the formulation with the least amount of leucine (5%) after two months of storage at 40 °C. This formulation was designed to favor interference of moxidectin with leucine crystallization. No fibers were observed in the other formulations until the 8-month mark, when a small number of fibers could be detected only in the formulation with 30% leucine stored at 40 °C. The formulation with 50% leucine was designed to allow leucine to crystallize first without interference from moxidectin. This formulation showed no fiber formation. As presented in Table 5, leucine was detected to be mostly in the crystalline phase in all the formulations. Very small fractions of amorphous leucine would have remained undetected, due to the detection limit of the instrument. Leucine resembled the β polymorph in the formulations with 30% and 50% leucine. The polymorphism of the 5% powder could not be reliably determined.

Lastly, no leucine fiber formation was observed in the mannitol formulations. In these formulations, both excipients crystallized without leaving a measurable amorphous fraction behind. Leucine was present in the most stable α polymorph in all three formulations containing mannitol. This polymorph is encountered when leucine crystallizes by itself, e.g., in the raw material. Thus, its presence indicated that there was minimal interaction between leucine and mannitol crystallization in this system, regardless of the sequence.

These results taken together show that leucine fiber formation in long-term storage occurs in situations that allow the substantial interference of other excipients or actives with the crystallization of leucine. In these cases, it is likely that small amounts of disordered leucine phases with higher energy states were formed that had a tendency to relax over the course of the stability challenge if given the sufficient mobility. Importantly, the mass fraction of L-leucine that formed these fibers was negligible compared to the bulk of the leucine fraction, which remained stable irrespective of the solid phase it was in. The results demonstrate that fiber formation can be minimized or avoided altogether if formulations are designed using particle formation models in such a way as to give leucine ample time to crystallize before other components of the system precipitate.

## 4. Conclusions

A stability study of leucine in conjunction with three classes of pharmaceutical actives confirmed that microstructures resembling thin fibers may appear in leucine-containing spray-dried powders during storage. The formation of these fibers was correlated with small fractions of disordered leucine caused by the interference of other components with the crystallization of leucine. If desired, the frequency of the appearance of these fibers can be reduced via careful formulation design by giving leucine the sufficient time to crystallize, consequently minimizing the amorphous content. Furthermore, it was concluded that the appearance of these fibers does not adversely affect the key quality attributes applicable to dry powder inhalers, such as aerosol performance and physicochemical stability. The overall findings indicate that leucine remains an attractive excipient candidate for dispersibility enhancement, even in cases where a small amount of fibers is formed during storage.

## Figures and Tables

**Table 1:** The selected formulations based on the results obtained from the particle formation models and the predicted outcome. $t_{w,\mathrm{leu}}$ is the predicted crystallization window of leucine defined as the time at which the next component reached its respective critical concentration on the droplet surface minus the time at which leucine reached the critical concentration on the surface.

Formulation Name	Total Solids Loading (mg/mL)	Mass Fractions (-)			$t_{w,leu}*$ (ms)	Predicted Outcome
First System		Bevacizumab	Trehalose	Leucine		
B18T72L10	40	0.18	0.72	0.10	−5.5	Fully amorphous leucine
B13T52L35		0.13	0.52	0.35	0.4	Partially amorphous leucine
B10T40L50		0.10	0.40	0.50	3.1	Fully crystalline leucine
**Second System**		**Ethanol:Water**	**Moxidectin**	**Leucine**		
Mx95L5	5	0.5:0.5	0.95	0.05	-	Least leucine crystallinity
Mx70L30	10		0.70	0.30	-	Moderate leucine crystallinity
Mx50L50	10		0.50	0.50	-	Most leucine crystallinity
**Third System**			**Mannitol**	**Leucine**		
Mn90L10	40		0.90	0.10	−1.8	Mannitol nucleates first
Mn81L19			0.81	0.19	0.0	Both nucleate together
Mn60L40			0.60	0.40	3.7	Leucine nucleates first

* $t_{w,\mathrm{leu}}$ is the predicted crystallization window of leucine defined as the time at which the next component reached its respective critical concentration on the droplet surface minus the time at which leucine reached the critical concentration on the surface.

**Table 2:** The time points and assays for the stability analysis performed on the three model systems stored at 25 and 40 °C.

	SEM/Raman Spectroscopy	PXRD/NGI/APS/KF/DSC
Beva/Trehalose/Leucine	t_0_, 1, and 2 weeks, 1, 2, 4, and 8 months	t_0_, 4 months
Moxidectin/Leucine	t_0_, 2 weeks, 1, 2, 4, and 8 months	t_0_, 4 months
Mannitol/Leucine	t_0_, 1, and 2 weeks, 1, 2, 4, and 8 months	t_0_, 4 months

**Table 3:** The solid phases of the spray-dried formulations containing bevacizumab, trehalose, and leucine obtained from the deconvolution of their respective Raman spectra. The solid phases of all powders were stable for up to 8 months of storage at temperatures as high as 40 °C.

Formulation	t_0_		4 and 8 Months	
	mAb + Trehalose	Leucine	mAb + Trehalose	Leucine
B18T72L10	amorphous	amorphous	amorphous	amorphous
B13T52L35	amorphous	β-form	amorphous	β-form
B10T40L50	amorphous	β-form	amorphous	β-form

**Table 4:** The glass transition temperatures, water content, and active concentration values of the spray-dried formulations containing bevacizumab, trehalose, and leucine. The uncertainties represent one standard deviation of at least three replicates.

	B18T72L10			B13T52L35			B10T40L50		
	t_0_	4 Months at 25 °C	4 Months at 40 °C	t_0_	4 Months at 25 °C	4 Months at 40 °C	t_0_	4 Months at 25 °C	4 Months at 40 °C
Dry $T_{g}$ (midpoint, °C)	118.0 ± 0.2	119.1 ± 0.4	118.3 ± 2.1	121.5 ± 2.0	123.9 ± 0.4	123.4 ± 0.7	122.0 ± 3.2	121.7 ± 1.4	121.8 ± 0.5
Water Content (wt %)	4.82 ± 0.09	4.23 ± 0.09	3.75 ± 0.04	3.59 ± 0.00	2.81 ± 0.11	2.86 ± 0.08	2.92 ± 0.01	2.44 ± 0.00	2.23 ± 0.08
mAb Concentration (wt %)	19.1 ± 0.9	18.1 ± 0.1	18.2 ± 0.3	13.0 ± 0.1	13.0 ± 0.1	12.7 ± 0.1	9.2 ± 0.2	9.6 ± 0.1	9.0 ± 0.1

**Table 5:** The solid phases of the spray-dried formulations containing moxidectin and leucine obtained from the deconvolution of their respective Raman spectra. The solid phases of all powders were stable for up to 8 months of storage at temperatures as high as 40 °C.

Formulation	t_0_		4 and 8 Months	
	Moxidectin	Leucine	Moxidectin	Leucine
Mx95L5	amorphous	Crystalline *	amorphous	Crystalline *
Mx70L30	amorphous	β-form	amorphous	β-form
Mx50L50	amorphous	β-form	amorphous	β-form

* The polymorphic form could not be determined.

**Table 6:** The glass transition temperatures, water content, and active concentration values of the spray-dried moxidectin–leucine formulations. The uncertainties represent one standard deviation of at least three replicates.

	Mx95L5			Mx70L30			Mx50L50		
	t_0_	4 Months at 25 °C	4 Months at 40 °C	t_0_	4 Months at 25 °C	4 Months at 40 °C	t_0_	4 Months at 25 °C	4 Months at 40 °C
Dry $T_{g}$ (midpoint, °C)	115.9 ± 1.9	114.0 ± 1.0	116.7 ± 0.3	115.2 ± 0.7	116.8 ± 0.3	115.8 ± 0.5	117.9 ± 2.1	115.4 ± 1.2	119.6 ± 0.1
Water Content (wt %)	0.66 ± 0.02	0.46 ± 0.02	0.42 ± 0.01	0.58 ± 0.01	0.44 ± 0.02	0.40 ± 0.03	0.49 ± 0.02	0.38 ± 0.05	0.35 ± 0.01
Moxidectin Concentration (wt %)	95.6 ± 0.8	95.0 ± 0.8	94.8 ± 0.6	70.7 ± 0.2	70.3 ± 0.2	69.6 ± 0.5	50.8 ± 0.1	50.4 ± 0.1	50.2 ± 0.1

**Table 7:** The melting temperatures, water content, and active concentration values of the spray-dried formulations containing mannitol and leucine. The uncertainties represent one standard deviation of at least three replicates.

	Mn90L10			Mn81L19			Mn60L40		
	t_0_	4 Months at 25 °C	4 Months at 40 °C	t_0_	4 Months at 25 °C	4 Months at 40 °C	t_0_	4 Months at 25 °C	4 Months at 40 °C
$T_{m}\;$ (Onset, °C)	164.5 ± 0.1	164.8 ± 0.1	164.8 ± 0.1	163.7 ± 1.2	164.3 ± 0.0	164.3 ± 0.1	164.4 ± 0.0	164.4 ± 0.1	164.4 ± 0.0
Water Content (wt %)	0.04	0.02	0.04	0.04	0.09	0.09	<0.01	0.03	<0.01
Mannitol Concentration (wt %)	90.9	90.7	91.6	83.0	82.5	83.0	62.5	63.8	63.4

**Table 8:** The prevalence of leucine fibers in electron micrographs of the three model systems during 8 months of storage under two different conditions. The time points correspond to the number of months of storage.

Formulation	t_0_	t_0.5_		t_1_		t_2_		t_4_		t_8_	
		25 °C	40 °C	25 °C	40 °C	25 °C	40 °C	25 °C	40 °C	25 °C	40 °C
B18T72L10	0	+	++	+	+++	++	+++	++	+++	+++	+++
B13T52L35	0	0	+	+	++	+	+++	+	+++	+	+++
B10T40L50	0	0	+	0	+	0	+	0	+	0	++
Mx95L5	0	0	0	0	0	0	+	0	++	+	+++
Mx70L30	0	0	0	0	0	0	0	0	0	0	+
Mx50L50	0	0	0	0	0	0	0	0	0	0	0
All mannitol formulations	0	0	0	0	0	0	0	0	0	0	0

Legend: 0 no fibers, + scarce short fibers, ++ prevalent short fibers, and +++ many long fibers.

## Data Availability

Not applicable.
